# Antibody Delivery into the Brain by Radiosensitizer Nanoparticles for Targeted Glioblastoma Therapy

**DOI:** 10.3390/jnt3040012

**Published:** 2022-09-30

**Authors:** Omer Gal, Oshra Betzer, Liat Rousso-Noori, Tamar Sadan, Menachem Motiei, Maxim Nikitin, Dinorah Friedmann-Morvinski, Rachela Popovtzer, Aron Popovtzer

**Affiliations:** 1 Davidoff Cancer Center, Rabin Medical Center, Beilinson Hospital, Petach Tikva 4941492, Israel; 2 Faculty of Engineering, Institute of Nanotechnology & Advanced Materials, Bar-Ilan University, Ramat Gan 5290002, Israel; 3 School of Neurobiology, Biochemistry and Biophysics, George S. Wise Faculty of Life Sciences, Tel Aviv University, Tel Aviv 6997801, Israel; 4 Moscow Institute of Physics and Technology, MIPT, Dolgoprudny, 141701 Moscow, Russia; 5 Department of Nanobiomedicine, Sirius University of Science and Technology, 354340 Sochi, Russia; 6 Sagol School of Neuroscience, Tel Aviv University, Tel Aviv 6997801, Israel; 7 Sharett Institute of Oncology, Hadassah Medical Center, Hebrew University of Jerusalem, Jerusalem 91120, Israel

**Keywords:** glioblastoma, gold nanoparticles, cetuximab, blood-brain barrier, radiotherapy

## Abstract

**Background:**

Glioblastoma is the most lethal primary brain malignancy in adults. Standard of care treatment, consisting of temozolomide (TMZ) and adjuvant radiotherapy (RT), mostly does not prevent local recurrence. The inability of drugs to enter the brain, in particular antibody-based drugs and radiosensitizers, is a crucial limitation to effective glioblastoma therapy.

**Methods:**

Here, we developed a combined strategy using radiosensitizer gold nanoparticles coated with insulin to cross the blood-brain barrier and shuttle tumor-targeting antibodies (cetuximab) into the brain.

**Results:**

Following intravenous injection to an orthotopic glioblastoma mouse model, the nanoparticles specifically accumulated within the tumor. Combining targeted nanoparticle injection with TMZ and RT standard of care significantly inhibited tumor growth and extended survival, as compared to standard of care alone. Histological analysis of tumors showed that the combined treatment eradicated tumor cells, and decreased tumor vascularization, proliferation, and repair.

**Conclusions:**

Our findings demonstrate radiosensitizer nanoparticles that effectively deliver antibodies into the brain, target the tumor, and effectively improve standard of care treatment outcome in glioblastoma.

## 1 Introduction

Glioblastoma is the most common primary brain malignancy in adults, with a dismal prognosis [1]. Despite an improvement in standard of care treatment with temozolomide (TMZ) and radiotherapy (RT) [2], local recurrence rates remain high, and median life expectancy remains low [3]. This largely results from tumor radioresistance, mediated by such factors as tumor stem cells and hypoxia [4,5], as well as from poor drug penetration due to the restrictive blood-brain barrier (BBB). Strategies to overcome radioresistance, such as radiation dose escalation [6], hyperfractionation schedules with higher total doses [7], or stereotactic radiosurgery [8], have shown no benefit in randomized trials. Novel radiosensitizers, and numerous other experimental glioblastoma drugs tested in clinical trials, have unfortunately all failed [9–18]. Although TMZ can also serve as a radiosensitizer, it is subject to rapid hydrolysis, while also causing damage to healthy cells, as it is not tumor-specific.

Effective treatment of glioblastoma is hindered by the BBB, which prevents brain uptake of most drugs, including radiosensitizers, and tumor-specific drugs—in particular antibody-based therapies. Though glioblastoma can show sporadic increase in BBB permeability, therapeutic agents must cross intact BBB regions to access the entire tumor [19–21]. Therefore, there is a crucial need for agents that have capability to overcome the BBB, target the tumor, and enable tumor radiosensitizaton.

Nanoparticles are rapidly becoming impactful biomedical delivery tools, providing functionality, biocompatibility, and therapeutic precision. In particular, gold nanoparticles (GNPs) are considered ideal radiosensitizing agents, due to their biocompatibility, and high absorption and enhancement of ionizing radiation [22–24]. GNPs have unique physiochemical properties, allowing easy tuning of size and conjugation to various biomolecules for active tumor targeting [25–29]. Our group and others have shown that antibody conjugation actively targets GNPs to various solid tumors, and effectively potentiates radiotherapy [24,30–32]. By the concentration of targeted nanoparticles within the tumor, its absorbed portion of incident radiation energy increases, while reducing damage to surrounding tissue [22]. Yet safe and efficient entry of tumor-targeted antibodies into the brain, either as free molecules or conjugated to nanoparticles, has not yet been shown.

We have previously shown that coating GNPs with insulin enables their crossing of the BBB [33,34]. Here, we developed GNPs that cross the BBB and shuttle anti-epidermal growth factor receptor (EGFR) antibodies into the brain, to actively target the commonly amplified EGFR in glioblastoma. Intravenously injected GNPs coated with insulin and cetuximab (CTX-INS-GNPs) showed successful crossing of the BBB and high accumulation within an orthotopic glioblastoma in mice. Moreover, combining standard of care with these targeted GNPs effectively eradicated tumor cells, blocked tumor growth, and enhanced survival of the mice.

## 2 Materials and Methods

### 2.1 Synthesis and Characterization of CTX-INS-GNPs

Synthesis of 20 nm spherical GNPs was carried out using sodium citrate as a reducing agent, based on Enüstün and Turkevic’s methodology [35]. Briefly, 414 μL of 50% *w/v* HAuCl4 solution was added to 200 mL purified water, and the solution was heated in an oil bath on a heating plate until boiling. Then, 4.04 mL of 10% sodium citrate solution was added, and the solution was stirred for 10 min. After cooling to room temperature, the solution was centrifuged until precipitation of the nanoparticles. GNPs were coated with mPEG-SH (60%) and SH-PEG-COOH (5000 Da (20%)) (Creative PEGWorks, Winston Salem, NC, USA), and stirred for three hours. The carboxylic group of SH-PEG-COOH was covalently conjugated to human insulin (Novo Nordisk A/S, Bagsvaerd, Denmark; 1.5 mL, 100 IU/mL) by activation with EDC (1-Ethyl-3-(3-dimethylaminopropyl)) carbodiimide HC1 (EDC, 200 μL, Thermo Scientific, Waltham, MA, USA) and N-Hydroxysulfosuccinimide sodium salt (NHS, 200 μL, Thermo Scientific, Waltham, MA, USA) and then centrifuged. Next, SH-PEG-COOH (3500 Da (20%)) was added for binding of CTX (Erbitux, Merck KGaA, Darmstadt, Germany) by activation with EDC-NHS. The mixture was subsequently stirred overnight in ice. Centrifugation (4 °C) was performed until a final Au concentration of 30 mg mL—1 was reached.

Transmission electron microscopy (TEM, JEM-1400, JEOL, Tokyo, Japan) was used to measure the size and shape of the GNPs, which were further characterized using ultraviolet-visible spectroscopy (UV-Vis; UV-1650 PC; Shimadzu Corporation, Kyoto, Japan) and zeta potential (ZetaSizer 3000HS; Malvern Instruments, Malvern, UK), following each level of coating.

### 2.2 Animal Experiments

All animal experiments and procedures were approved by the Animal Care Committee of the University Health Network and performed in accordance with the National Institutes of Health guidelines and regulations. Animals were monitored for clinical signs (changes in skin and fur, eyes, nose, mouth, locomotion); blood samples were collected (200 mL) before, one week after, and four weeks after injection, under general anesthesia by retro-orbital sinus bleeding for analysis of complete blood count, liver function, and renal function (creatinine, urea, liver transaminases).

Tumor induction, CT imaging, and radiation therapy were carried out under general anesthesia. Mice were sacrificed when clinical deterioration was observed, or at the end of the study protocol (180 days after tumor induction).

### 2.3 Orthotopic Glioblastoma Xenografts

Athymic nude mice (male; 8 weeks) were injected intracranially with human U87 cells (3 × 10^4^), at 2 mm posterior and 1.5 mm lateral to the bregma. Fourteen days after induction, tumor development was verified and tumor size was measured pre-treatment, using CT scan (below).

### 2.4 Treatment of Mice

On day 14 after tumor induction, the orthotopic glioblastoma tumor-bearing mice were randomly divided into groups: a group of mice treated with standard of care TMZ and RT, consisting of intraperitoneal TMZ (10 mg/kg for 5 days) and fractionated 6 MV X-ray irradiation to the whole brain (10 Gy in 5 days; 2 Gy/day) (*n* = 10); a group of mice treated with TMZ and RT (as detailed above), together with CTX-INS-GNPs (intravenously; 0.006 g GNP with 3.7 mg/kg CTX per 200 μL injection) (π = 8); and an untreated group (*n* = 5). We note that as CTX alone, or in combination with RT and TMZ, does not demonstrate efficacy, as widely demonstrated in preclinical and clinical studies [36,37], this treatment was not included in the study.

Weekly CT imaging (clinical CT, LightSpeed VCT, GE) was performed to measure tumor size and characteristics; regions of interest (ROIs) were manually drawn covering the entire tumor region, and tumor size was defined as the maximal 3D diameter measured.

### 2.5 Micro-CT Scans

In vivo scans for detection of the GNPs in brains were performed using a micro-CT scanner (Bruker, Sky scan high-resolution model 1176, Kontich, Belgium) with a nominal resolution of 35 μm, a 0.2 mm aluminum filter, and a tube voltage of 40 kV. Reconstruction was carried out with a modified Feldkamp algorithm using the SkyScan NRecon software (Bruker Skyscan NRecon VI.7.4.2, Kontich, Belgium) accelerated by GPU. Ring artifact reduction, Gaussian smoothing (3%), and beam hardening correction (20%) were applied. Volume rendered 3D images were generated using an RGBA transfer function in SkyScan CT-Voxel (Bruker CTvox V3.3.l, 3D.SUITE software, Kontich, Belgium) software.

### 2.6 Immunohistochemistry

Mouse brains were extracted (at the experiment conclusion at 180 days after treatment, or after clinical deterioration) and immediately placed in formaldehyde and later embedded in paraffin. Five μm consecutive sections in triplicate slides were prepared from four areas within each brain (*n* = 3/group). Each 2nd slide from each area was stained for hematoxylin and eosin (H&E) for tumor presence verification and localization. Immunohistochemical ⋂uorescence (IHC-F) staining was performed on the 1st and 3rd slides from one tumor-containing area. Sections were de-paraffinized and epitope retrieval was performed, and then incubated for 1 h with primary antibodies (1:50, mouse monoclonal anti-mouse PCNA #307902, Biolegend + 1:50, rabbit monoclonal anti-mouse EGFR Ab52894, Abeam; 1:50, rat monoclonal anti-CD34 Ab8l58, Abeam + 1:50, rabbit monoclonal anti-mouse Ki67 #275R-14, Cell Marque) followed by incubation with secondary antibodies (1:200, donkey anti-mouse Cy2 715-545-151, Jackson, USA+ 1:200, donkey anti-rabbit Cy3 711-165-152, Jackson, USA; 1:200, donkey anti-rat Cy3 712-165-153, Jackson, USA+ 1:200, donkey antirabbit Cy2 712-225-152, Jackson, USA; respectively). Slides were then stained with nuclei marker 4′6-Diamidino-2-P henylindole, Dilactate (DAPI, 1:400, BLG-422801, Biolegend, USA) and covered. Images were obtained by using a Leica TCS SP5 confocal laser-scanning microscope (Leica Microsystems, Wetzlar, Germany). All photos for specific staining were taken in the same exposure conditions; hence, signal intensity is comparable between groups and samples. Staining with secondary antibodies only served as negative control for immunofluorescence staining and was used for background reduction.

### 2.7 Inductively Coupled Plasma-Optical Emission Spectrometry (ICP-OES) Analysis

To measure brain accumulation of the CTX-INS-GNPs, brains were extracted, post perfusion, 6 h after IV injection, and gold concentrations were measured using ICP-OES (710, Agilent Technologies, Santa Clara, CA, USA). Samples were dissolved in aqua regia acid (a mixture of nitriac acid and hydrochloric acid in a volume ratio of 1:3), the acid was evaporated by heating, and the samples diluted with purified water to a total volume of 5 mL. After filtration of the samples, gold concentrations were determined according to absorbance values, with correlation with calibration curves, constructed from solution with known gold concentrations (0, 0.5, 2, and 5 mg/L).

### 2.8 Statistical Analysis

Data were analyzed using the SPSS statistical software (IBM ® SPSS ® Statistics Version 25, Armonk, NY, USA) at a significance level of 0.05. Relative tumor growth was analyzed by two-way repeated measures ANOVA. Survival was assessed by Kaplan-Meier survival analysis and compared using the log-rank test.

## 3 Results

### 3.1 GNP Characterization

**Figure 1:**
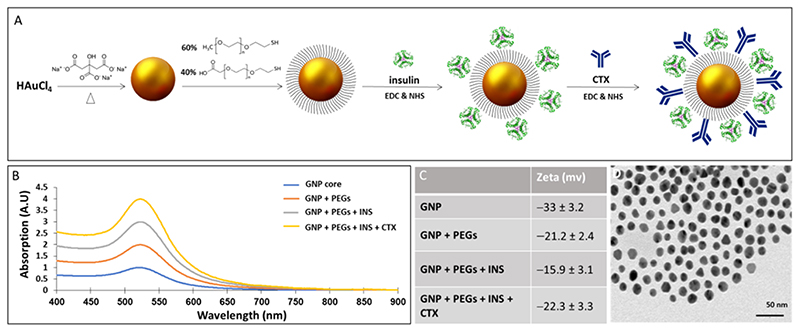
Synthesis and characterization of CTX-INS-GNPs. (A) Scheme of CTX-INS-GNP synthesis; (B) Ultraviolet-visible spectroscopy of the synthesis stages: bare GNPs, PEG-coated GNPs, INS-GNPs, and CTX-INS-GNPs, showing expanded and shifted signal after each layer of coating; (C) Zeta potential measurements of the synthesis stages of CTX-INS-GNPs. The clear differences obtained following each chemical step demonstrate the efficiency of the coating stages; (D) Transmission electron microscopy image of the nanoparticles (Scale bar 50 nm). CTX: Cetuximab; EDC: N-(3-dimethylaminopropylj-N’-ethylcarbodiimide; GNPs: Gold nanoparticles; INS: Insulin; NHS: N-hydroxysuccinimide; PEG: Polyethylene Glycol.

GNPs sized 20 nm were prepared and covalently coated with insulin and CTX (Figure 1A). Characterization of the nanoparticles with transmission electron microscopy showed uniform, spherical GNPs, with a mean size of ~2O nm in diameter. UV-vis plasmon resonance shift and expansion and zeta potential measurements confirmed the subsequent coating layers (Figure 1B-D). An in vitro cell binding experiment verified the specificity and targeting ability of the GNPs toward EGFR (Figure SI, Supplementary Material).

### 3.2 CTX-INS-GNPs Combined with Standard Therapy Inhibits Tumor Progression and Prolongs Survival

We have previously demonstrated that coating of GNPs with insulin enables their crossing of the BBB [33,34]. Here, we found that insulin-coated GNPs conjugated with antibodies to their surface, and injected intravenously to mice, retain the ability to cross the BBB and reach brain regions (Figure S2, Supplementary Material).

**Figure 2:**
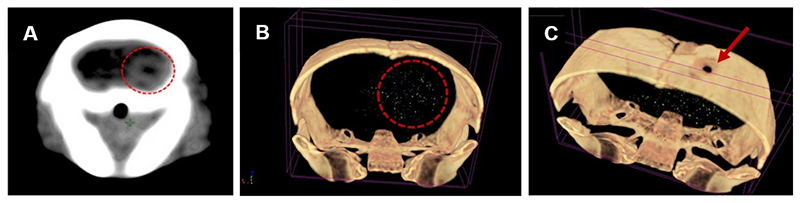
CT scans confirming tumor establishment and CTX-INS-GNPs within it. (A) Clinical CT scan of mouse brain 14 days after tumor induction shows tumor establishment in the brain (denoted by red circle). (B) 3D volume rendered micro-CT scan of mouse brain, showing that the CTX-INS-GNPs (gold dots) reached and accumulated at the tumor site (denoted by red circle). (C) Site of injection of the glioblastoma cells at the top of the skull can also be seen, as indicated by the red arrow.

Next, we investigated the effect of combining CTX-INS-GNPs together with standard therapy on glioblastoma tumor progression and mouse survival. An orthotopic tumor was induced in mice by intracranial injection of human U87 cells (3 × 10^4^). CT scans performed 14 days later confirmed tumor establishment in mice, with an average maximal diameter of 2.3 mm (Figure 2A). The tumor-bearing mice were then either left untreated (*n* = 5) or treated with TMZ + RT (*n* = 10), or with intravenously injected CTX-INS-GNPs together with TMZ and RT (*n* = 8), and tumors were measured over six weeks.

The CT scans performed one week after treatment demonstrated that CTX-INS-GNPs successfully crossed the BBB and accumulated within the tumor (Figure 2B,C). Elemental ICP-OES analysis of gold content in the brain showed 0.0338 ± 0.009 mg CTX-INS-GNPs in brain tissue, a high brain uptake of CTX that is at least ~I5 fold higher than free antibody uptake in the brain [38,39].

**Figure 3:**
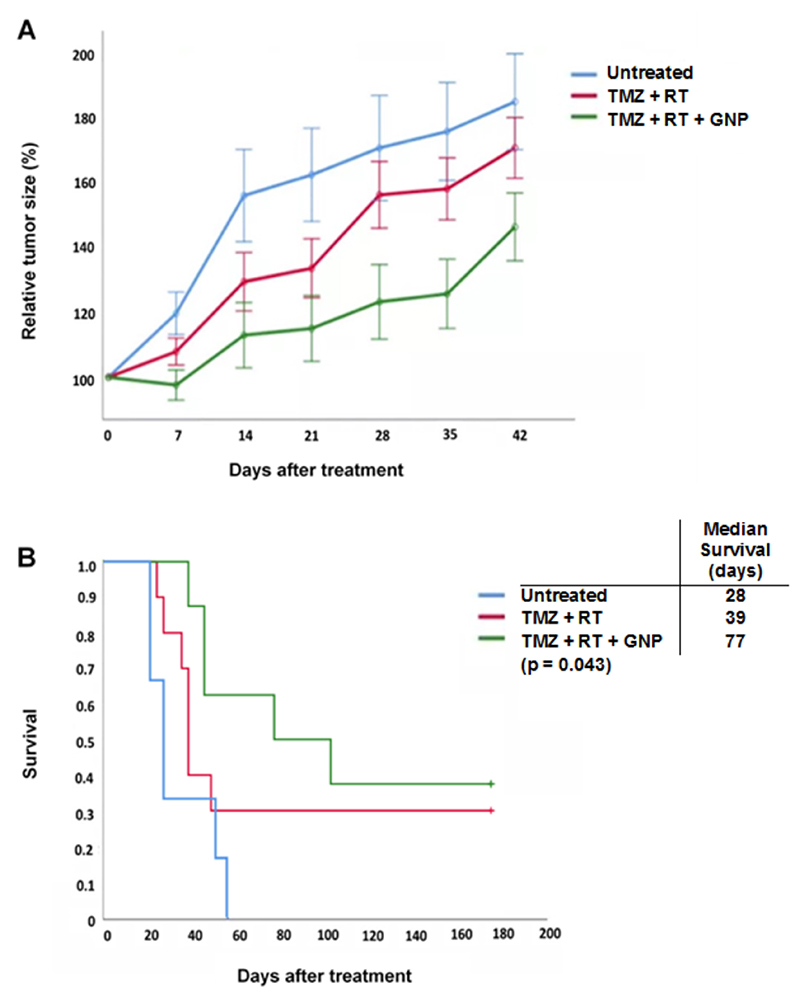
CTX-INX-GNP combined with standard of care chemotherapy and radiotherapy inhibited tumor growth and prolonged survival of mice with orthotopic glioblastoma. (A) In mice bearing orthotopic glioblastoma tumors, treatment combining CTX-INS-GNP with TMZ and RT significantly inhibited tumor growth up to six weeks after treatment, as compared to untreated mice or mice treated with TMZ + RT. Two-way ANOVA with repeated measures showed a main effect of group (F(2,19) = 4.37; *p* < 0.028). Results presented as mean ±SEM. (B) Kaplan-Meier survival curve of mice with orthotopic glioblastoma. Addition of CTX-INS-GNPs to standard of care therapy significantly increased median survival (*p* = 0.043, Kaplan-Meier log-rank test) as compared to untreated controls and standard-of-care (RT + TMZ) treated mice.

Weekly CT imaging was performed to measure tumor size from the day of treatment up to six weeks later. We found that treatment with CTX-INS-GNPs together with TMZ and RT led to significant inhibition of tumor growth over six weeks after treatment, as compared to mice treated with TMZ + RT and untreated mice (two-way ANOVA with repeated measures, *p* < 0.028; Figure 3A). Moreover, survival was assessed up to 180 days after tumor induction. Mice treated with CTX-INS-GNP combined with TMZ and RT showed significantly extended median survival (77 days), as compared to standard TMZ + RT treatment (39 days) and untreated control (28 days) (*p* = 0.043, Kaplan-Meier log rank test; Figure 3B).

### 3.3 Combined Treatment with CTX-INS-GNPs Eradicates Tumor Cells

**Figure 4:**
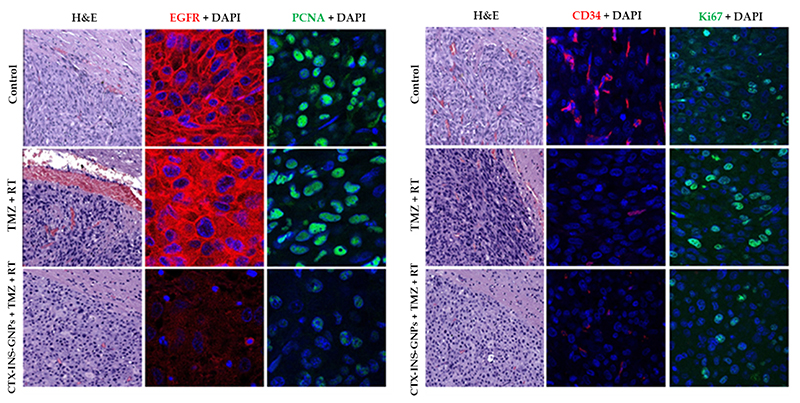
Histological characterization of treated tumors. Representative images of tumor sections after IHC-F staining, for untreated mice, mice treated with TMZ + RT, or mice treated with CTX-INS-GNPs combined with TMZ and RT, at day 42 after treatment. Left image: Sections were stained with H&E; anti-EGFR (red) and DAPI (blue), showing lower EGFR expression in the GNP-treated group; and with PCNA (green) and DAPI, showing reduced DNA repair in the GNP-treated group. Right image: Tumor sections were stained with CD34 (red), indicating lower angiogenesis in the RT + TMZ with or without GNPs as compared to control, and stained with Ki67 (green) indicating lower proliferation of tumor cells after RT + TMZ with GNPs. X4O magnification.

Ex-vivo histological analysis was performed to further investigate the effect of CTX-INS-GNPs combined with RT and TMZ on glioblastoma tumors. H&E staining showed tumor cells in brain sections of all study groups. In mice that were found dead during the study, most brains were necrotic, and tumor tissue could not be distinguished from normal brain. IHC-F staining showed complete elimination of EGFR in the group treated with CTX-INS-GNP combined with TMZ + RT, as compared to the untreated control and standard-of-care treatment groups. This indicates that the CTX-INS-GNPs indeed targeted EGFR-expressing tumor cells, which enabled total elimination of these cells. CD34 staining levels were lower in both the CTX-INS-GNP-treated group and the standard-of-care-treated group as compared to the untreated control, indicating that the treatments decreased tumor angiogenesis. PCNA and Ki-67 staining were lower in the CTX-INS-GNP-treated group than in the other two groups, indicating that the addition of the nanoparticles to standard of care therapy reduced tumor proliferation and tissue repair levels (Figure 4 and Figure S3).

During the study period, no skin toxicity or behavioral changes were detected in the animals; moreover, blood count, white blood cell hemoglobin and platelets, and blood chemistry parameters were within normal ranges in treated animals, indicating the biocompatibility of the nanoparticles.

## 4 Discussion

In the present study, we demonstrated the efficacy of radiosensitizer GNPs for shuttling an antibody across the BBB and actively targeting the tumor, thus enhancing standard of care therapy for glioblastoma. CTX-INS-GNPs successfully crossed the BBB and specifically accumulated within the tumor. Combining these GNPs with conventional RT and TMZ significantly inhibited tumor growth and prolonged survival. Histological analysis further revealed that the combined therapy reduced tumor proliferation and repair, and eradicated EGFR-expressing tumor cells.

Targeted radiosensitizers have potential to increase tumor sensitivity to radiation while reducing healthy tissue toxicity, thereby increasing the therapeutic window. The dismal prognosis of glioblastoma due to local recurrence makes it an ideal candidate for the addition of targeted radiosensitizers to standard of care treatment. However, a wide range of novel therapeutic approaches studied in clinical trials have failed to yield viable radiosensitizers. A main reason for these failures is the inability of radiosensitizers to penetrate the restrictive BBB [40]. The BBB can be sporadically disrupted in glioblastoma, due to rapid tumor neovascularization and altered protein expression [41], which can lead to enhanced permeability and retention (EPR) of nanoparticles in tumors. However, the EPR effect in glioblastoma is inefficient, due to a dense brain matrix impeding diffusion, and the elevated interstitial fluid pressure. Additionally, therapeutic agents must cross intact BBB regions to access the entire tumor [19–21].

A few studies have developed nanoparticles that cross the BBB, yet this was mediated via cumbersome external apparatuses that are capable of destabilizing, or even potentially damaging, the BBB [42–44]. Insulin traverses the BBB via receptor-mediated transcytosis, and we have previously shown that insulin coating of GNPs for targeting insulin receptors enables crossing of the BBB and high accumulation in the brain, as compared to non-coated GNPs [34]. Here, we show for the first time that our insulin-coated GNPs retain this BBB-crossing ability even after additional antibody conjugation, and effectively shuttle a glioblastoma-targeting antibody across the BBB. The INS-GNP-bound CTX had high brain uptake, approx. 15 fold more than the uptake of free antibody [38,39]. Furthermore, CTX-INS-GNP selectively targeted the tumor and accumulated within, which led to complete elimination of EGFR-expressing tumor cells.

High-Z metal nanoparticles such as GNPs can enhance the therapeutic ratio of radiation therapy, by augmenting the effective dose within tissues. The proposed mechanism for this effect is the increased secondary electron and free radical production in the tumor microenvironment, which amplify the radiobiological effects on DNA [45]. GNPs are the most studied, and among the most potent, metallic nanomaterials for radiation enhancement. More than two decades ago, Regulla et. al. [46] showed a 160-fold higher efficacy in X-ray irradiation-induced killing of cells grown upon a gold monolayer, as compared to those grown on plastic. This was due to enhanced release of electrons, which create ionization and free radicals, thus more radiation dose is deposited locally around the gold. Hainfeld et. al. [22] demonstrated that GNPs delivered to mammary carcinomas and then irradiated resulted in much greater tumor destruction than radiation alone. Previous studies have shown that GNPs coated with poly-allylamine, and functionalized with CTX, selectively target EGFR-overexpressing head and neck cancer cells [47], have a radiosensitizing effect on these cells in vitro [48], and in mice show only limited and transient toxicity [49]. In addition, our group has previously designed CTX-bound GNPs, and showed that following intravenous injection, these GNPs actively and efficiently targeted head-and-neck tumor xenografts in mice [50,51], and enhanced the RT effect, which significantly inhibited tumor growth, and reduced tumor vascularity, proliferation, and tissue repair, with no toxicity to healthy tissue [24]. Other studies [52–55] have shown encouraging results regarding the ability of GNPs to be effective radiosensitizers for GBM treatment; however, these GNPs did not deliver antibodies across the BBB for specific tumor targeting. Here, we demonstrated that adding cetuximab and insulin-coated GNPs to standard of care therapy led to considerable damage to glioblastoma tumor tissue, and decreased its vascularization, proliferation, and repair.

Taken together, our findings using GNPs coated with insulin and a targeting antibody can have a large impact on facilitating delivery of targeting agents to glioblastoma, and lead to effective radiosensitization. However, it should be noted that this study included only one glioblastoma cell line model, and although it clearly proves the principle that the CTX-INS-GNPs are able to cross the BBB and target the tumor, these results need to be confirmed in future studies with additional glioblastoma cell lines and patient-derived xenografts models.

As glioblastoma continues to be a fatal disease, novel treatment approaches are keenly awaited. The present study emphasizes the importance of investigations of additional promising targeting moieties. For instance, future research may consider to further improve selectivity towards EGFR-expressing glioblastoma tumors, by specific targeting of the extracellular domain mutation EGRvIII. Importantly, the growing body of evidence showing the diverse, and effective, use of GNPs in different clinical scenarios calls for considering translational studies to test these opportunities for novel glioblastoma treatment approaches.

## 5 Conclusions

In conclusion, this study demonstrated a novel approach, combining the radiosen-sitizing properties of GNPs together with the BBB-crossing properties of insulin and the tumor-targeting properties of CTX, which effectively improved treatment outcomes in mice carrying intracranial glioblastoma. These BBB-crossing and actively targeted GNP have further potential for delivery of various therapeutics that may be effective in combating glioblastoma.

## Supplementary Material

Supplementary data

## Data Availability

The datasets used and/or analyzed during the current study are available from the corresponding author on reasonable request.
