# Supplementary material for: Antibody Delivery into the Brain by Radiosensitizer Nanoparticles for Targeted Glioblastoma Therapy
Source: J Nanotheranostics. Author manuscript; Available in PMC 2022 Nov 1. (PMC7613745; doi:10.3390/jnt3040012)
Supplement: Supplementary data [file EMS155543-supplement-Supplementary_data.pdf]

Supplementary Data

Antibody Delivery into the Brain by Radiosensitizer Nanoparticles for Targeted Glioblastoma Therapy

Omer Gal [1], Oshra Betzer [2], Liat Rousso-Noori [3], Tamar Sadan [2], Menachem Motiei [2], Maxim Nikitin [4], Dinorah Friedmann-Morvinski [3,5], Rachela Popovtzer [2] and Aron Popovtzer [6,*]

1 Davidoff Cancer Center, Rabin Medical Center, Beilinson Hospital, Petach Tikva 4941492, Israel

2 Faculty of Engineering and the Institute of Nanotechnology & Advanced Materials Bar-Ilan University, Ramat Gan, 5290002, Israel

3 School of Neurobiology, Biochemistry and Biophysics, George S. Wise Faculty of Life Sciences, Tel Aviv University, Tel Aviv 6997801, Israel

4 Moscow Institute of Physics and Technology, MIPT, Dolgoprudny, Russia

5 Sagol School of Neuroscience, and School of Neurobiology, Tel Aviv University, Tel Aviv 6997801, Israel

6 Sharett Institute of Oncology, Hadassah Medical Center, Hebrew University of Jerusalem, Jerusalem, Israel

* Correspondence: aron@hadassah.org.il; Tel.: 972-2-6777825

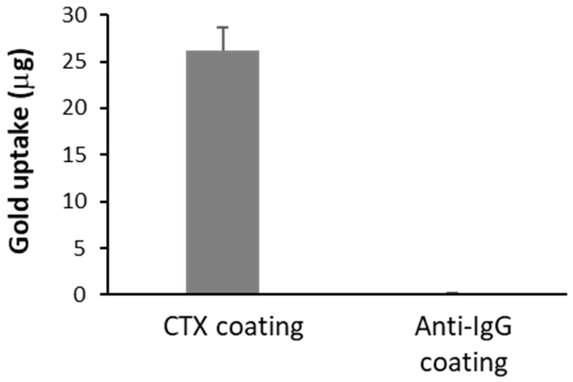

Figure S1. To verify the targeting ability of GNPs coupled to CTX toward EGFR, the particles coupled to either CTX or to a nonspecific antibody (anti-rabbit IgG) were incubated with cancer cells that highly express EGFR (A431 cells, 2.5×10^6) for 30 minutes at 37 °C. Quantitative atomic absorption spectroscopy measurements demonstrated a significantly higher gold quantity absorbed by cells treated with the targeted CTX-coated GNPs as compared to those treated with the non-specific anti-IgG-coated GNPs.

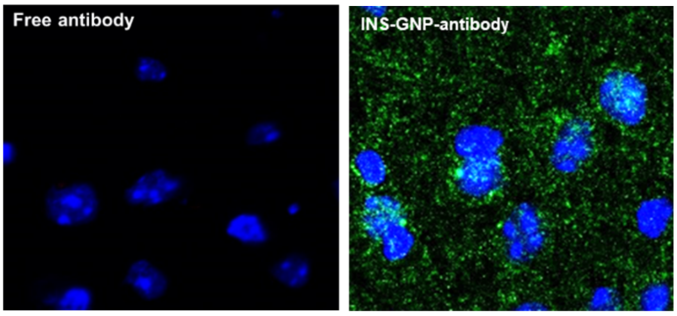

Figure S2. Insulin coating of GNPs successfully shuttles antibodies across the BBB: Mice received IV injection of either free antibody (IgG) or insulin-coated GNPs conjugated with the antibody and brains were excised 24 h later. Immunofluorescent imaging of brain sections showed that free antibody did not enter the brain, while the insulin-coated GNPs successfully delivered the conjugated antibody (INS-GNP-antibody; green) into brain regions (shown is stained cerebral cortex). Blue: DAPI nuclei staining, green: antibody. Images obtained with confocal laser-scanning microscope; magnification 63×.

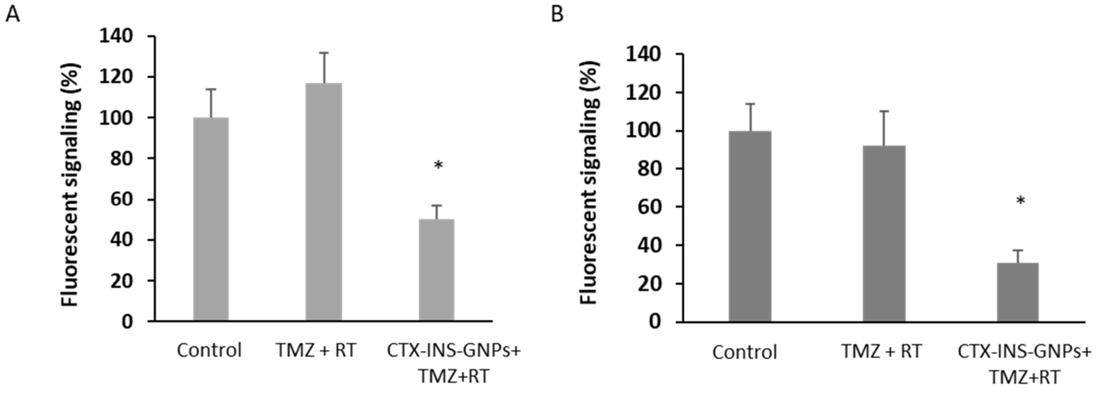

Figure S3. Quantification of fluorescent signaling in brain section images stained with KI67 (**A**) or PCNA (**B**) of untreated controls, TMZ + RT-treated mice, and mice treated with CTX-INS-GNP combined with RT and TMZ; analyzed with ImageJ (normalized to control expressed as 100%). * $p < 0.05$ for the combined treatment vs. standard of care and untreated.

Methods

Evaluation of targeting of EGFR: For synthesis of the GNPs with an anti-rabbit IgG coating, the anti-IgG antibody (Jackson ImmunoResearch Laboratories, Inc, West Grove, PA) was bound to the particles via SH-PEG-COOH by activation with EDC-NHS. Successful synthesis of the particles was confirmed via ultraviolet-visible spectroscopy (UV-1650 PC; Shimadzu Corporation, Kyoto, Japan) and zeta potential (ZetaSizer 3000HS; Malvern Instruments, Malvern, UK).

Next, high EGFR expressing A431 cancer cells (2.5×10^6; Dulbecco's modified Eagle's medium (5 mL) with 5% FCS, 0.5% glutamine, and 0.5% penicillin) were incubated with either the CTX-coated or anti-rabbit IgG-coated particles at 50 μL; 25 mg/mL, for 30 minutes at 37 °C. After incubation, the medium was washed twice with PBS and aqua regia (1 mL) was added. After acid evaporation, the sediment was dissolved in hydrochloride (5 mL 0.05 M). Gold concentrations were quantified by atomic absorption spectroscopy (AA 140; Agilent Technologies, Santa Clara, CA). Each group was run in triplicate.

Immunocytochemistry fluorescence imaging of brains: Mice received IV injection of either free IgG or insulin-coated GNPs conjugated to IgG (mouse monoclonal IgG1 Alexa Fluor 488 Isotype Control Clone 11711). Twenty-four hrs later, brains were excised and frozen in chilled 2-methyl butane (Sigma-Aldrich), stored at 4 °C, and subsequently sectioned into slices (7 μm), stained with DAPI, and fluorescence was detected and imaged with Leica TCS SP5 confocal laser-scanning microscope (Leica Microsystems, Germany), as previously described [1].

Reference

1. Perets, N; Betzer, O; Shapira, R; Brenstein, S; Angel, A; Sadan, T; Ashery, U; Popovtzer, R; Offen, D. Golden Exosomes Selectively Target Brain Pathologies in Neurodegenerative and Neurodevelopmental Disorders. *Nano Letters* **2019**, *19*, 3422–3431.
